# A High-Density Genetic Linkage Map of SLAFs and QTL Analysis of Grain Size and Weight in Barley (*Hordeum vulgare* L.)

**DOI:** 10.3389/fpls.2020.620922

**Published:** 2020-12-17

**Authors:** Yunxia Fang, Xiaoqin Zhang, Xian Zhang, Tao Tong, Ziling Zhang, Gengwei Wu, Linlin Hou, Junjun Zheng, Chunyu Niu, Jia Li, Wenjia Wang, Hua Wang, Dawei Xue

**Affiliations:** ^1^College of Life and Environmental Sciences, Hangzhou Normal University, Hangzhou, China; ^2^State Key Laboratory for Managing Biotic and Chemical Threats to the Quality and Safety of Agro-Products, Institute of Virology and Biotechnology, Zhejiang Academy of Agricultural Sciences, Hangzhou, China

**Keywords:** barley, genetic linkage map, grain size, QTL, SLAF markers

## Abstract

Grain size is an important agronomic trait determines yield in barley, and a high-density genetic map is helpful to accurately detect quantitative trait loci (QTLs) related to grain traits. Using specific-locus amplified fragment sequencing (SLAF-seq) technology, a high-density genetic map was constructed with a population of 134 recombinant inbred lines (RILs) deriving from a cross between Golden Promise (GP) and H602, which contained 12,635 SLAFs with 26,693 SNPs, and spanned 896.74 cM with an average interval of 0.07 cM on seven chromosomes. Based on the map, a total of 16 QTLs for grain length (GL), grain width and thousand-grain weight were detected on 1H, 2H, 4H, 5H, and 6H. Among them, a major QTL locus *qGL1*, accounting for the max phenotypic variance of 16.7% was located on 1H, which is a new unreported QTL affecting GL. In addition, the other two QTLs, *qGL5* and *qTGW5*, accounting for the max phenotypic variances of 20.7 and 21.1%, respectively, were identified in the same region, and sequencing results showed they are identical to *HvDep1* gene. These results indicate that it is a feasible approach to construct a high-quality genetic map for QTL mapping by using SLAF markers, and the detected major QTLs *qGL1*, *qGL5*, and *qTGW5* are useful for marker-assisted selection (MAS) of grain size in barley breeding.

## Introduction

Barley is one of the most important cereal crops in the world, and widely used for animal feed and malting (Baik and Ullrich, 2008; Bond et al., 2015). Previous genome sequencing projects had indicated that the barley has a genome of 5.1 Gb, which is much larger than human genome of 3.3 Gb and rice genome of 389 Mb (International Barley Genome Sequencing Consortium et al., 2012). Due to the high repetitive sequences and complex structure, the sequence assembly of barley genome had been affected greatly, and the accuracy and completeness of the physical map need to be further improved (Mascher et al., 2017). Although barley is also a diploid species, the numbers of genes have been cloned are far less than rice and Arabidopsis, and the reverse genetics is usually used to carry out gene function studies (Fu et al., 2007; Sikdar et al., 2016; Holme et al., 2017). Therefore, it has become a common strategy to identify quantitative trait loci (QTLs) for important agronomic traits for marker-assisted selection (MAS) in barley (Romagosa et al., 1999; Miedaner and Korzun, 2012; Zhang et al., 2017; Fang et al., 2019).

Quantitative trait loci mapping has been widely used to identify genomic regions associated with target trait, which mainly subject to the sample size and molecular marker density (Doerge and Rebai, 1996). Due to the low density polymorphism of traditional molecular markers over the whole genome, the precise of QTL mapping was greatly limited (McCouch et al., 1988, 2002; Olson et al., 1989). With the development of high-throughput genotyping and sequencing technology, the massive single nucleotide polymorphisms (SNPs) were extensively identified in different species, which usually used for high-density map construction, genome-wide association analysis, gene mapping, gene chip, MAS, etc. (Wang et al., 1998; Slate et al., 2009; Huang et al., 2010; Shao et al., 2015; Li et al., 2018). However, whole genome deep re-sequencing is still costly and not necessary for most studies. So, reduced representation genome sequencing (RRGS) was developed by DNA fragments sequencing of restriction enzyme digestion, which exhibits the advantages in identifying and genotyping SNPs, including simple steps, high effectivity, low cost, short cycle, and so on (Van Tassell et al., 2008; Hyten et al., 2010). Among them, specific-locus amplified fragment sequencing (SLAF-seq) is one version of RRGS based on special fragment-length, which mainly applied in high-density genetic map construction and gene mapping in many species (Zhang et al., 2013; Li et al., 2014; Hu et al., 2016; Yu et al., 2019; Zhao et al., 2019).

Grain weight and size are two important agronomic traits that determine barley yield and malt quality, and easily influenced by the environment, in which grain weight is defined as the sum of thousand-grain weight (TGW), and grain size is constituted by grain length (GL), grain width (GW), and grain thickness (GT) (Coventry et al., 2003; Watt et al., 2019). Recent studies have identified a number of genes involved in grain weight and size in barley, such as *HvDep1*, *Nud, D-hordein*, *Vrs1*, *Vrs2*, *Vrs3, Vrs4*, and *Vrs5/Int-c* (Taketa et al., 2008; Ramsay et al., 2011; Koppolu et al., 2013; Wendt et al., 2016; Bull et al., 2017; Sakuma et al., 2017; Youssef et al., 2017; Yang et al., 2020). Moreover, multiple important QTLs for grain weight and size have also been identified. Among them, the major QTLs for kernel length, *LEN-3H*, *LEN-4H*, *14LEN-6H*, and *14LEN-7H* were mapped on 3H, 4H, 6H, and 7H, which accounted for the phenotypic variances of 29.1, 16.4, 17.6, and 17.2%, respectively (Zhou et al., 2016). Both of GW and length QTLs, *QTL-GW1*, and *QTL-GT1* were located on 5H, and explained the max phenotypic variances of 13.9 and 19.0%, respectively (Watt et al., 2019). Two grain weight QTLs were also identified, and exhibited 88 and 12.6% phenotypic variances (Kjaer and Jensen, 1996; Thomas et al., 1998). Using a DH population, a grain volume QTL was identified on 2H with the max phenotypic variance of 19.3%, and three GL QTLs were mapped on 2H, 2H and 5H with the max phenotypic variances of 24.7, 23.3, and 22.6%, respectively (Walker et al., 2013).

In the present study, a total of 12,635 SLAF markers with 26,693 SNPs were employed to genotype the recombinant inbred lines (RILs) derived from a cross between H602 and Golden Promise (GP), and a high-density genetic map spanned 896.74 cM was constructed. The QTL analysis of grain size and weight was subsequently performed, and three major QTLs *qTGW5*, *qGL1*, and *qGL5* were identified with the max phenotypic variances of 21.1, 16.7, and 20.7%, respectively. The results will accelerate the QTL mapping of important agronomic trait loci and facilitate the MAS of grain size in barley.

## Materials and Methods

### Plant Materials and DNA Extraction

A RILs population of F8 generation was constructed via single seed descent, which derived from the cross of GP and H602 (a wild barley strain). The parents and developed 134 RILs were planted in the experimental fields of Hangzhou Normal University, Hangzhou, Zhejiang province (120°20 E, 30°27 N) with conventional field cultivation (row spacing of 20 cm). After harvest and drying in 2017–2019, the TGW, GL and GW were measured using an SC-G automatic seed analyzer (WSeen, China, *n* > 50). Total genomic DNAs of young healthy leaves were extracted from parents and 134 lines by CTAB method with some modification (Doyle and Doyle, 1987). The full-length genomic DNA of *HvDep1* gene was amplified and sequenced with primers HvDep1-1, HvDep1-2, HvDep1-3, and HvDep1-4, respectively, which were listed in the Supplementary Table 1.

### SLAF Library Construction and High-Throughput Sequencing

Specific-locus amplified fragment library construction was carried out following the description in detail by Sun et al. (2013) and the 5.1G barley genome was used as a reference genome. The Genome DNAs of parents and 134 RILs were digested by *Hae*III (New England Biolabs, NEB, United States) restriction enzyme, and a single nucleotide (A) overhang was subsequently added to the obtained fragment. Then, through sequencing adapters ligating, polymerase chain reaction (PCR) amplifying, Agencourt AMPure XP beads (Beckman Coulter, High Wycombe, United Kingdom) purifying, pooling, 2% agarose gel electrophoresing, and the fragments ranging from 364–414 bp were collected and purified by a QIAquick gel extraction kit (Qiagen, Hilden, Germany). Through the Illumina HiSeqTM 2500 platform (Illumina, Inc.; San Diego, CA, United States), the finally products sequencing was carried out at Biomarker Technologies Corporation (Beijing, China).

### SLAF-Seq Data Grouping and Genotyping

The reads obtained from sequencing were further distinguished and qualified reads with quality score more than 20e were distributed into each progeny based on duplex barcode sequence. According to over 90% sequence similarity, the reads were blasted by one to one alignment and the sequences clustered into the same group were defined as one SLAF. Based on the parental sequence depth more than 10×, the genotype of each SLAF marker was determined, which contained no less than 30% progeny information. Because barley is a diploid species, and one polymorphic SLAF marker can contain two to four alleles in the progenies, so more than four were got rid of as repetitive SLAFs. To genotype the polymorphic SLAF, parental genotypes were first determined, and then the offspring were also defined according to the consistency of the sequence with the parent. However, RIL is a permanent homozygous population, only the polymorphic SLAFs with the segregation type of aa × bb were adopted.

### Construction of High-Density Genetic Map

According to the standard of modified logarithm of odds (MLOD), the polymorphic SLAF markers were classified and partitioned primarily into seven linkage groups (LGs) by the position of barley reference genome. Due to the massive SNP data, the HighMap software was employed to construct high-density genetic map (Liu et al., 2014), in which the genotyping errors were corrected, the linear arrays of markers were ordered, and the genetic distances between two adjacent markers were computed by Kosambi mapping function in each LG. To ensure the quality of genetic map, the colinearity analysis between genetic maps and barley genomes were also evaluated.

### Data and QTL Analysis

To reduce errors, all measurement values were gained from average value of three biological replicates. The frequency distributions of grain weight and size were analyzed in the 3 years, and statistical and correlation analysis were performed with SPSS 20.0 software. Based on the high-density genetic map, a powerful software qgene-4.3.10 (Joehanes and Nelson, 2008) was adopted to carry out QTL analysis to identify the related locus. The composite interval mapping (CIM) model was used to scan the whole seven chromosomes by the interval of one milliMorgan. When logarithm of odds (LOD) threshold more than 3, the statistical significance (*P* = 0.05) was considered, and the target interval was determined again with 1,000 permutations. The QTL parameters of chromosomes, marker names and intervals, Generalized *R*^2^, LOD values and additive effects were computed by qgene-4.3.10.

## Results

### Analysis of SLAF-Seq Data and SLAF Markers

**FIGURE 1:**
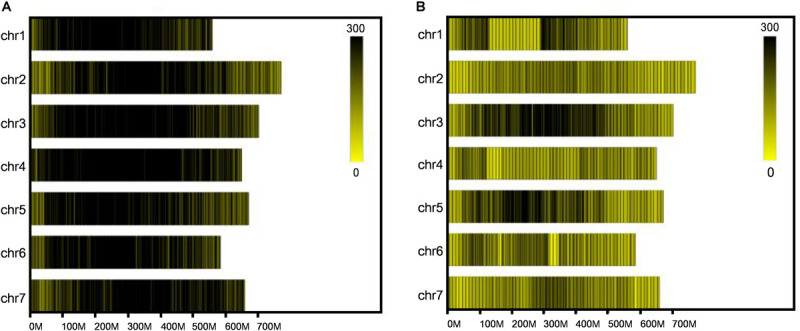
Distribution of total SLAFs and polymorphic SLAFs on each chromosome. **(A)** Distribution of total SLAF markers on each chromosome. **(B)** Distribution of polymorphic SLAF markers on each chromosome. *X*-axes represent the length of chromosomes, and yellow bands represent chromosomes. Barley genome was divided according to the size of 1 Mb, and each black line represents a SLAF marker.

After SLAF library construction and high-throughput sequencing, 231.56 Gb data containing 1158.81 M reads was obtained. The average Q30 ratio of all samples was 94.87%, and guanine-cytosine (GC) content was 47.81% in average (Table 1), which indicated that the data quality is qualified. All the reads were filtered and then aligned by blast software, and more than 90% similarity were defined as one SLAF. A total of 746,752 SLAFs were developed and divided into polymorphic, non-polymorphic and repetitive types, in which 245,618 polymorphic SLAF markers were identified, accounting for 32.95% of the total SLAFs (Figure 1 and Supplementary Table 2). Then, the 245,618 SLAFs were further classified into eight segregation patterns, and only aa × bb class was suitable for RIL population (Supplementary Figure 1 and Supplementary Table 3). Finally, 99,182 polymorphic markers fell into aa × bb class, which was applied for genetic map construction.

**TABLE 1:** Statistical analysis of sequencing data for each sample.

**Sample ID**	**Total reads**	**Total bases**	**Q30 percentage (%)**	**GC percentage (%)**
H602	27,352,189	5,468,336,348	94.00	47.20
GP	30,530,507	6,102,905,786	93.81	47.19
offspring	8,155,003	1,629,571,751	94.89	47.82
Total	1,158,808,151	231,563,428,464	94.87	47.81

### Construction of High-Density Genetic Linkage Map

**FIGURE 2:**
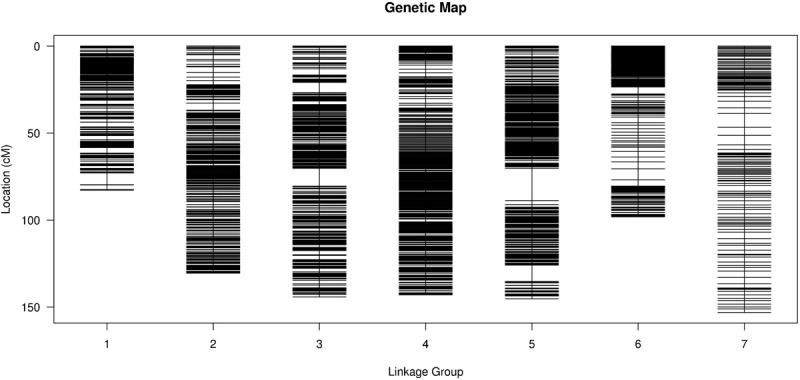
Distribution of SLAF markers on each chromosome. *X*-axis indicates the chromosomes of barley, and *Y*-axis indicates genetic distance of each chromosome. Black lines represent SLAF markers.

To improve the quality of genetic map and accuracy of QTL detection, the polymorphic SLAFs were screened again, and the retained SLAF markers were compared with reference genome of barley in order to observe the distribution of markers on each chromosome. Ultimately, 12,635 SLAF markers with 26,693 SNPs were mapped to seven LGs by HighMap software with average coverage depth 82.04-fold in GP, 76.57-fold in H602, and 23.36-fold in offspring (Figure 2 and Table 2^1^). The total genetic distance of linkage map was 896.74 cM with an average distance of 0.07 cM between adjacent markers. The number of markers in each LG varied from 154 to 6,109, and the genetic length of each LG differed from 82.85 to 153.06 cM. The degree of linkage between markers was reflected by “Gap < 5” ranging between 98.69 and 100% with an average value of 99.77%, and the largest gap was mapped on chromosome 5H with 18.47 cM (Table 3).

**TABLE 2:** Sequencing depth of retained SLAF markers.

**Sample ID**	**SLAF number**	**Total depth (X)**	**Average depth (X)**
GP	12,635	1,036,549	82.04
H602	12,635	967,406	76.57
Offspring	12,482	291,564	23.36

**TABLE 3:** Information statistics of genetic linkage map.

**LG ID**	**SLAFs**	**SNPs**	**Total distance (cM)**	**Average distance (cM)**	**Gap < 5 cM**	**Max Gap (cM)**	**Spearman**
chr1	1,396	3,114	82.85	0.06	99.93%	6.82	0.995
chr2	710	1,725	130.41	0.18	100.00%	4.04	0.994
chr3	6,109	12,588	144.20	0.02	99.97%	10.18	0.921
chr4	1,011	2,342	142.94	0.14	100.00%	2.76	0.997
chr5	1,678	3,536	145.18	0.09	99.88%	18.47	0.986
chr6	1,577	3,045	98.10	0.06	99.94%	6.34	0.940
chr7	154	343	153.06	1.00	98.69%	7.98	0.995
Total	12,635	26,693	896.74	0.07	99.77%	18.47	/

*LG, linkage group; SLAFs, the total SLAF markers on each LG; SNPs, the numbers of SNP markers; Total distance, the total genetic distance of each LG; Average distance, the average distance between two flank markers; Gap < 5 cM, the proportion of gap less than 5 cM; Spearman, correlation coefficient between each LG and the physical graph, and the closer the values to 1, the better the collinearity between the two maps.*

### Grain Weight and Size Data Analysis

**FIGURE 3:**
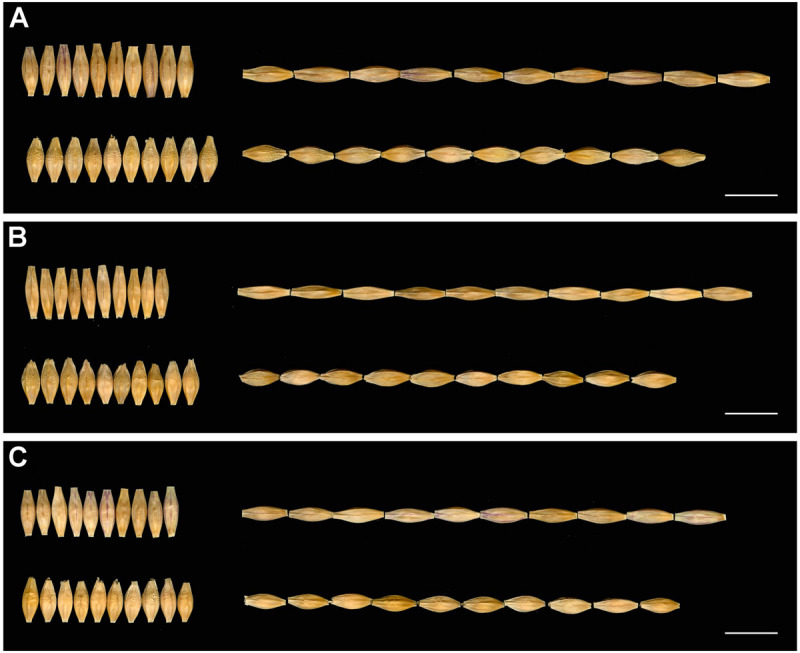
Seeds of H602 and GP in 3 years. **(A–C)** The seeds of H602 and GP in 2017–2019. The top of each figure is H602, and the bottom is GP. Scale bars = 1 cm.

**FIGURE 4:**
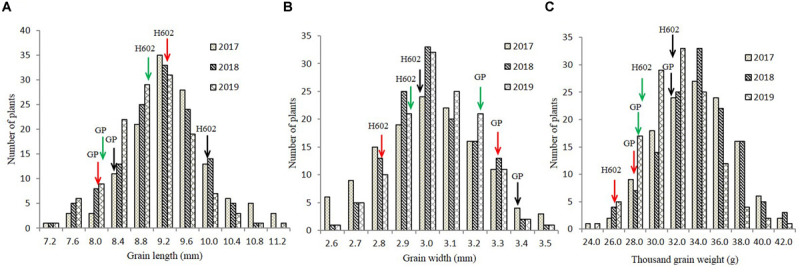
Frequency distribution of grain length, grain width, and thousand-grain weight in field trials. **(A–C)** The distribution of grain length, grain width, and thousand-grain weight in 2017–2019. *X*-axes represent the grain length, grain width, and thousand-grain weight, and *Y*-axes represent the numbers of plant at a certain length, width, or weight. Black, red, and green arrows indicate phenotypic values of GP and H602 in 2017–2019.

The GL, GW, and TGW of the parents and 134 RILs planted in Hangzhou were measured in consecutively 3 years, and the data showed that parent GP exhibited decreased GL and increased GW than parent H602 (Figure 3 and Supplementary Table 4). Moreover, the average GL and width of 134 RILs in 3 years were all fell in between two parents, and average grain weight in 2018 and 2019 was higher than the two parents (Supplementary Table 4). It is well know that grain weight is mainly determined by grain size, so their correlations should be positively correlated. Consistent with it, the correlation coefficients between GL versus TGW, and GW versus TGW exhibited positive correlation (*P* < 0.01) (Supplementary Table 5). In addition, the frequency distributions of grain size and weight of 134 individuals were also analyzed, and the results indicated that data were normally distributed, and suitable for QTL mapping (Figure 4).

### QTL Mapping of Grain Size and Thousand-Grain Weight

**FIGURE 5:**
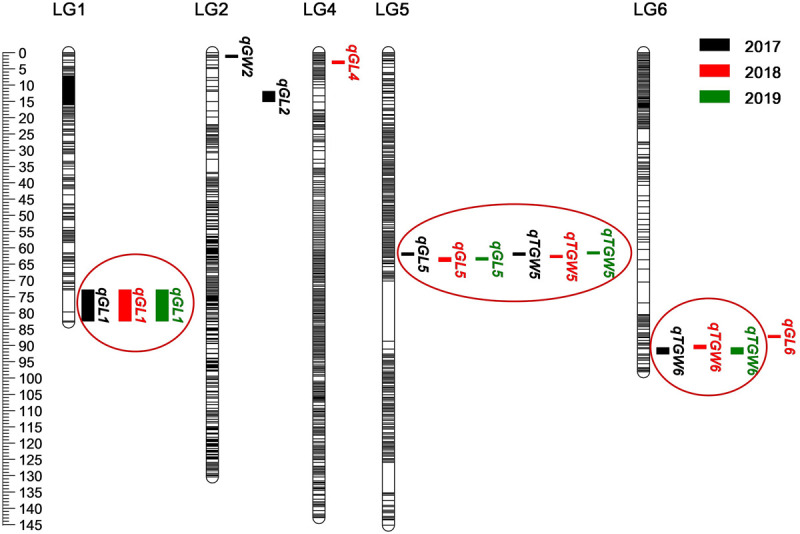
Location of 16 QTLs for grain weight and size on genetic linkage map. The black, red, and green boxes indicate QTLs detected in 2017–2019, respectively. Black lines represent SLAF markers.

Based on the high-density genetic map, a total of 16 QTLs related to grain size and TGW were detected on chromosome 1, 2, 4, 5, 6H in consecutive 3 years, and accounted for phenotypic variances ranged from 10.2 to 21.1% (Figure 5 and Table 4). Among them, three QTLs for GL, one QTL for GW, and two QTLs for TGW were located in 2017, four QTLs for GL and two QTLs for TGW were mapped in 2018, and two QTLs for GL and two QTLs for TGW were detected in 2019. The four QTLs, *qGL1*, *qGL5*, *qTGW5*, and *qTGW6* were repeatedly detected in 3 years, in which *qGL1* explained the phenotypic variance ranged from 14.9 to 16.7%, *qGL5* from 17.6 to 20.7%, *qTGW5* from 17.9 to 21.1%, and *qTGW6* from 10.9 to 12.0%. Meanwhile, the average GL, width and weight of 3 years were also used to detect QTL, and the results indicated that the located QTLs and its intervals were similar to single-year data, except for *qGL6* (Supplementary Table 6). In addition, the positions of *qGL5* and *qTGW5* were located in the same interval or neighboring to each other in 3 years, suggesting the two QTLs may be the same locus.

**TABLE 4:** QTLs identified in the RIL population for grain size and weight.

**Year**	**QTL**	**Chr**	**Pos. (cM)**	**Peak LOD**	***R*^2^ (%)**	**Effect**	**Flank markers**	**Physical pos. (Mb)**
2017	qTGW5	5	61.9	6.89	21.1	–0.164	Marker4410393-Marker6905115	481.70–482.01
	qTGW6	6	91	3.71	12.0	0.124	Marker3078489-Marker3057930	575.92–577.84
	qGL1	1	76.1	5.33	16.7	–0.321	Marker17833427-Marker15481727	524.12–535.28
	qGL2	2	14.6	3.36	10.9	–0.245	Marker23989565-Marker26402843	641.73–644.16
	qGL5	5	61.9	6.76	20.7	–0.331	Marker4410393-Marker6905115	481.70–482.01
	qGW2	2	1.1	3.67	11.8	–0.071	Marker25707515-Marker23519662	143.99–266.11
2018	qTGW5	5	62.6	5.73	17.9	–0.141	Marker6905115-Marker6734745	482.01–483.63
	qTGW6	6	90.6	3.37	10.9	0.11	Marker1722076-Marker2479549	575.35–576.58
	qGL1	1	78.5	5.05	15.9	–0.28	Marker17833427-Marker15481727	524.12–535.28
	qGL4	4	3	4.06	13.0	–0.242	Marker13166768-Marker11238390	32.15–44.41
	qGL5	5	63.8	6.59	20.3	–0.296	Marker3884634-Marker4645053	482.84–483.61
	qGL6	6	87.2	3.13	10.2	0.21	Marker1774327-Marker411944	571.95–573.68
2019	qTGW5	5	61.5	6.40	19.7	–0.133	Marker4941182-Marker6905115	481.44–482.01
	qTGW6	6	91	3.54	11.5	0.101	Marker3078489-Marker3057930	575.92–577.84
	qGL1	1	77	4.71	14.9	–0.279	Marker17833427-Marker15481727	524.12–535.28
	qGL5	5	63.6	5.64	17.6	–0.283	Marker3884634-Marker3656738	482.84–483.50

*Chr, chromosome ID; Pos., genetic position of QTL; Peak LOD, Maximum-likelihood LOD score; R^2^ (%), trait variation explained; Effect, locus effect, negative additive effect is from H602, and positive additive effect is from GP; Flank markers, flanking markers of detected QTL. Physical pos., position based on barley reference genome in Mb.*

### Candidate Regions of Major QTLs

**FIGURE 6:**
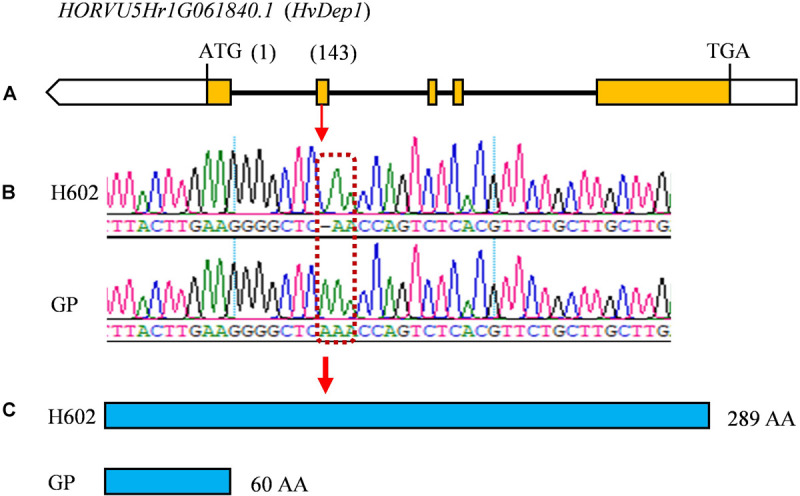
Differences in coding region of *HvDep1* in GP and H602. **(A)** Exons and introns of *HvDep1.* Yellow boxes represent the exons. **(B)** Differential locus of *HvDep1*in H602 and GP. **(C)** Protein sequence length of HvDep1 in two parents. Single base insertion of *HvDep1* in GP leads to transcription termination earlier.

Considering that the three major QTLs (*qGL1*, *qGL5*, and *qTGW5*) with high LOD scores and high phenotypic variations are valuable for further gene cloning and MAS in breeding, we carried out physical distances analysis of candidate regions. Among them, the major QTLs *qGL5* and *qTGW5* were finally located in a 2.18 Mb interval between Marker4941182 and Marker6734745. In the candidate region, *HvDep1*, a gene controlling grain weight and grain size was found (Wendt et al., 2016). The gene sequencing showed a single base insertion in the second exon of *HvDep1* was identified in parent GP, but there were no changes in parent H602 (Figure 6). Sequence analysis revealed that the gene was premature termination in GP, which is the same as reported *HvDep1* gene mutation (Wendt et al., 2016). So, *qGL5* and *qTGW5* should be the identical gene to *HvDep1*. In addition, another major QTL, *qGL1* was located in an 11.16 Mb region between Marker17833427 and Marker16397031 on chromosome 1H, and no related QTLs or genes have been reported in the interval.

## Discussion

In the modern breeding, MAS is significant to accelerate the selection process of target traits (Collard and Mackill, 2008; Bankole et al., 2017; Xu et al., 2018). However, important agronomic traits are usually controlled by QTL, and most of them have not been cloned or identified except for a few species. As an effective way for screening gene linkage, QTL analysis has been widely used to locate target trait gene and obtain linked markers, in which high-quality and high-density genetic map is a key for QTL mapping (Li et al., 2014; Wei et al., 2014). However, limited number of polymorphic markers is the main obstacle in the construction of high-density genetic map by traditional molecular markers (Hyten et al., 2010; Sun et al., 2013). SLAF-seq is an enhanced reduced representation library (RRL) sequencing technology with the advantage of massive SNP, high-resolution and low-cost, which has been successfully used to construct high-density linkage maps, gene mapping and association analysis (Wei et al., 2014; Li et al., 2014; Xia et al., 2015; Wen et al., 2020).

Due to large genome, it is difficult to develop enough traditional polymorphism markers for covering the genome uniformly, which results in a limited number of markers used to construct high-density genetic maps in barley. Using 1,000 SSR and DArT markers, a high-density genetic map was developed from a DH population, which spanned 1,100.1 cM and exhibited an average distance of 0.91 cM (Hearnden et al., 2007). Another high-density consensus map comprising 2,935 loci (2,085 DArT and 850 other loci) and spanning 1,161 cM was conducted, which derived from seven DH and three RIL populations, and showed an average inter-bin distance of 0.7 ± 1.0 cM (Wenzl et al., 2006). However, the two maps were not dense enough, and the average genetic distances were more than 0.7 cM. With the completion of barley genome sequencing in 2012, the SNP has become an important molecular marker for genetic analysis due to the massive single base-pair changes. Applying the RAD-seq strategy, 12,998 SNP markers were developed for the construction of high-density genetic map, which spanned 967.6 cM and displayed an average distance of 0.07 cM (Zhou et al., 2015). In 2017, a more high-quality barley reference genome was assembled and version IBSC_v2 was released, which increased the linear order of sequences and reduced the interference of repetitive elements (Mascher et al., 2017). In this study, we constructed a high-density genetic map of 12,635 SLAFs by the IBSC_v2 reference genome, which spanned 896.74 cM and exhibited an average distance of 0.07 cM. In the seven LGs, average value of “Gap < 5” reached 99.77%, and only two gaps larger than 10 cM were existed on chromosome 3H and 5H, respectively. So, the genetic map we constructed was high-quality and high-density, and suitable to conduct genetic analysis of important agronomic traits.

In order to verify the validity and accuracy of the map, the QTL analysis of grain size and weight was conducted, and a total of 16 QTLs related to GL, GW, and TGW were detected, in which four QTL loci, *qGL1*, *qGL5*, *qTGW5*, and *qTGW6* were repeatedly detected in 3 years. In view of the overlapped location interval, negative additive effects, and high correlation coefficients between GL and TGW, we speculated that *qGL5* and *qTGW5* should be the same QTL locus. Previous study showed that *qGL5H* was initially located in the position of 48.7–71.1 cM and fine mapped to a 1.7 Mb interval on chromosome 5, which situated in the 2.18 Mb section of *qGL5*/*qTGW5* (Watt et al., 2019). In the region, *HvDEP1*, an AGG3-type subunit of G protein encoding gene that regulating GL and TGW was also identified, which indicated that it might be the candidate gene of *qGL5*, *qGL5H*, and *qTGW5* (Wendt et al., 2016). The subsequent sequencing results showed that the same single base insertion was found in the CDS of parent GP, which revealed the identity of *qGL5*/*qTGW5* and *HvDEP1*. In addition, through different populations, several major QTLs for GL have been mapped on chromosome 2, 3, 4, 5, 6, and 7H, and no major QTL was found on chromosome 1H (Walker et al., 2013; Zhou et al., 2016; Watt et al., 2019, 2020). So, *qGL1* should be an unreported new QTL for GL. Although *qTGW6* only explained the max phenotypic variance of 12.0% for grain weight, the QTL was also repeatedly detected in 3 years, indicated that *qTGW6* is stably inherited.

## Conclusion

Grain size and weight are important agronomic traits determinant yield. In this study, we constructed a high-density genetic map with 12,635 SLAFs, and identified two major QTLs involved in regulating GL and grain weight. Among them, an unreported new QTL, *qGL1* accounted for maximum phenotypic variance of 16.7% and showed a negative additive effect on GL, which indicated the QTL from H602 played a promoting effect on elongating GL. Another major QTL locus, *qGL5*/*qTGW5* exhibited maximum phenotypic variance of 20.7 and 21.1% in GL and TGW, respectively, and also displayed negative additive effect, which revealed the QTL from GP reduced the GL and TGW. These results indicated the two QTLs, *qGL1* and *qGL5*/*qTGW5* are useful for MAS in accelerating the breeding process of barley grain size and weight.

## Data Availability Statement

The datasets generated for this study can be found in NCBI BioProject PRJNA673067, https://www.ncbi.nlm.nih.gov/bioproject/PRJNA673067/.

## Author Contributions

DX and HW designed the research, wrote the manuscript, and revised the manuscript. YF, XQZ, XZ, and TT performed the experiments, analyzed the data, and wrote the manuscript. ZZ, GW, LH, JZ, CN, HW, JL, and WW planted and collected the experimental materials. All authors contributed to the article and approved the submitted version.

## Conflict of Interest

The authors declare that the research was conducted in the absence of any commercial or financial relationships that could be construed as a potential conflict of interest.

## Footnotes

1 https://www.ncbi.nlm.nih.gov/sra/, accession number PRJNA673067
