# Supplementary material for: A High-Density Genetic Linkage Map of SLAFs and QTL Analysis of Grain Size and Weight in Barley (Hordeum vulgare L.)
Source: Front Plant Sci. 2020 Dec 17;11:620922. doi: 10.3389/fpls.2020.620922 (PMC7793689; doi:10.3389/fpls.2020.620922)
Supplement: Supplementary file 1 [file Table_1.DOCX]

**Supplementary table 1** Primers used for sequencing

| **Markers** | **Forward primers (5’-3’)** | **Reverse primers (5’-3’)** |
| --- | --- | --- |
| HvDep1-1 | GGCAAAGGCAAGAAGGACAAT | AATACTCCTACCTTGGATGCAGA |
| HvDep1-2 | TACGGTTAGGCTGCTCAATGC | GAAATTAAGGGACAGCTCTAGCCA |
| HvDep1-3 | CATGTCTGGAACTGCCTGTTTCT | GGCGGAACAAGAGCCACACT |
| HvDep1-4 | CTGCTTCAAGATCCCTTCGTG | CGTAACCATCTATTTGCTTACAACG |

**Supplementary table 2** Number of different types of SLAFs

| **Type** | **Polymorphic SLAFs** | **Non-Polymorphic SLAFs** | **Repetitive SLAFs** | **Total SLAFs** |
| --- | --- | --- | --- | --- |
| Number | 245,618 | 499,431 | 385 | 745,434 |
| Percentage | 32.95% | 67.00% | 0.05% | 100.00% |

**Supplementary table 3** Genotypes of segregated populations

| **Type** | **Paternal**  **Genotype** | **Maternal**  **Genotype** | **Offspring**  **Genotype** |
| --- | --- | --- | --- |
| abxcd | ab | cd | ac,ad,bc,bd,- |
| efxeg | ef | eg | ee,ef,eg,fg，-- |
| abxcc | ab | cc | ac,bc，-- |
| ccxab | cc | ab | ac,bc，-- |
| hkxhk | hk | hk | hh,hk,kk，-- |
| lmxll | lm | ll | lm,ll，-- |
| nnxnp | nn | np | nn,np，-- |
| aaxbb | aa | bb | F_2_(aa,ab,bb)，RIL/DH(aa,bb)-- |

**Supplementary table 4** Descriptive statistics of data in 2017-2019

| **Traits** | **GP**  **Mean±S** | **H602**  **Mean±SD** | **RIL lines** | | **Skewnees** | **Kurtosis** |
| --- | --- | --- | --- | --- | --- | --- |
|  |  |  | **Range** | **Mean±SD** |  |  |
| GL2017 | 8.27±0.28 | 9.91±0.51* | 7.09-11.19 | 9.12±0.74 | 0.073 | 0.701 |
| GL2018 | 8.01±0.11 | 9.10±0.10** | 7.15-10.45 | 8.92±0.67 | -0.391 | 0.297 |
| GL2019 | 7.99±0.2 | 8.80±0.10* | 7.18-11.06 | 8.75±0.69 | 0.290 | 0.620 |
| GW2017 | 3.39±0.11 | 2.98±0.09** | 2.50-3.47 | 2.96±0.21 | 0.028 | -0.512 |
| GW2018 | 3.26±0.06 | 2.74±0.12** | 2.55-3.42 | 2.98±0.17 | 0.213 | -0.342 |
| GW2019 | 3.18±0.06 | 2.90±0.06* | 2.58-3.41 | 2.99±0.17 | 0.026 | -0.150 |
| TGW2017 | 32.91±0.71 | 33.3±1.61 | 23.66-41.76 | 32.61±3.65 | 0.027 | -0.376 |
| TGW2018 | 27.69±0.75 | 26.03±1.02* | 25.35-41.68 | 32.84±3.43 | 0.064 | -0.216 |
| TGW2019 | 26.60±0.46 | 27.80±0.77 | 23.92-40.20 | 30.84±3.07 | 0.282 | 0.048 |

Mean ± SD, represents the means and the standard deviation. P-value was determined by student’s t-test. * and ** represent p-value < 0.05 and p-value <0.01.

**Supplementary table 5** Pearson’s correlation coefficients between grain size and grain weight

| **Trait** | **TGW2017** | **TGW2018** | **TGW2019** | **GL2017** | **GL2018** | **GL2019** | **GW2017** | **GW2018** |
| --- | --- | --- | --- | --- | --- | --- | --- | --- |
| TGW2018 | 0.721** |  |  |  |  |  |  |  |
| TGW2019 | 0.781** | 0.754** |  |  |  |  |  |  |
| GL2017 | 0.592** | 0.521** | 0.403** |  |  |  |  |  |
| GL2018 | 0.507** | 0.652** | 0.461** | 0.849** |  |  |  |  |
| GL2019 | 0.555** | 0.538** | 0.454** | 0.833** | 0.835** |  |  |  |
| GW2017 | 0.609** | 0.392** | 0.420** | 0.511** | 0.415** | 0.474** |  |  |
| GW2018 | 0.252** | 0.579** | 0.287** | 0.358** | 0.385** | 0.331** | 0.530** |  |
| GW2019 | 0.306** | 0.375** | 0.488** | 0.254** | 0.265** | 0.119 | 0.558** | 0.551** |

** indicates P<0.01.

**Supplementary table 6** QTLs identified for grain size and weigh with average data of three years

| **QTL** | **Chr.** | **Pos.**  **(cM)** | **Peak LOD** | **R^2^ (%)** | **Effect** | **Flank markers** | **Position (Mb)** |
| --- | --- | --- | --- | --- | --- | --- | --- |
| qTGW-5 | 5 | 61.5 | 7.67 | 23.2 | -0.145 | Marker4941182- Marker6232616 | 481.44- 482.48 |
| qTGW-6 | 6 | 91 | 4.29 | 13.7 | 0.112 | Marker3078489- Marker3057930 | 575.92- 577.84 |
| qGL-5 | 5 | 63.8 | 6.93 | 21.2 | -0.298 | Marker3884634- Marker4645053 | 482.84- 483.61 |
| qGL-4 | 4 | 3 | 3.431 | 11.1 | -0.22 | Marker13166768- Marker11238390 | 32.15- 44.41 |
| qGL-1 | 1 | 77.7 | 5.599 | 17.5 | -0.291 | Marker17833427- Marker18180869 | 524.12- 535.57 |
| qGW-2 | 2 | 1.6 | 3.404 | 11.0 | -0.151 | Marker25707515- Marker23519662 | 143.99-266.11 |

Chr, chromosome ID; Pos., genetic position of QTL; Peak LOD, Maximum-likelihood LOD score; R2(%), trait variation explained; Effect, negative additive effect is from H602, and positive additive effect is from GP; Flank markers, flanking markers of detected QTL. Physical pos., position based on barley reference genome in Mb


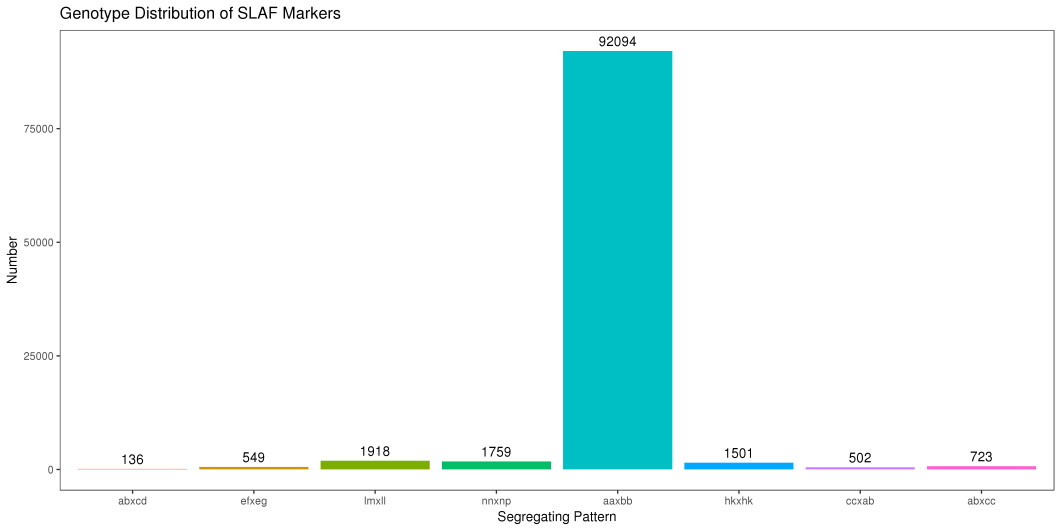


**Supplementary figure 1** Genotype distribution of SLAF markers

X-axis indicates segregating pattern, and y-axis indicates the number of different genotypes
